# Rapid conjugative mobilization of a 100 kb segment of *Bacillus subtilis* chromosomal DNA is mediated by a helper plasmid with no ability for self-transfer

**DOI:** 10.1186/s12934-017-0855-x

**Published:** 2018-01-27

**Authors:** Megumi Miyano, Kosei Tanaka, Shu Ishikawa, Shinji Takenaka, Andrés Miguel-Arribas, Wilfried J. J. Meijer, Ken-ichi Yoshida

**Affiliations:** 1 Department of Science, Technology and Innovation, Kobe University, 1-1 Rokkodai Nada, Kobe, 657 8501 Japan; 2 Organization of Advanced Science and Technology, Kobe University, 1-1 Rokkodai Nada, Kobe, 657 8501 Japan; 3 Department of Agrobioscience, Kobe University, 1-1 Rokkodai Nada, Kobe, 657 8501 Japan; 4 Centro de Biología Molecular ‘Severo Ochoa’ (CSIC-UAM), Instituto de Biología Molecular ‘Eladio Viñuela’ (CSIC), Universidad Autónoma, Canto Blanco, 28049 Madrid, Spain

**Keywords:** *Bacillus subtilis*, Conjugation, Gram positive, Plasmid transfer

## Abstract

**Background:**

The conjugative plasmid, pLS20, isolated from *Bacillus subtilis* natto, has an outstanding capacity for rapid self-transfer. In addition, it can function as a helper plasmid, mediating the mobilization of an independently replicating co-resident plasmid.

**Results:**

In this study, the *oriT* sequence of pLS20cat (*oriT*_LS20_) was eliminated to obtain the plasmid, pLS20catΔ*oriT*. This resulted in the complete loss of the conjugative transfer of the plasmid but still allowed it to mobilize a co-resident mobilizable plasmid. Moreover, pLS20catΔ*oriT* was able to mobilize longer DNA segments, up to 113 kb of chromosomal DNA containing *oriT*_LS20_, after mixing the liquid cultures of the donor and recipient for only 15 min.

**Conclusions:**

The chromosomal DNA mobilization mediated by pLS20catΔ*oriT* will allow us to develop a novel genetic tool for the rapid, easy, and repetitive mobilization of longer DNA segments into a recipient chromosome.

## Background

Bacterial cell division is asexual, and the genetic traits of mother and daughter cells with normal development are not changed fundamentally. However, horizontal gene transfer (HGT) can provide genetic plasticity for bacteria, even among different species [1]. HGT can lead sometimes to problematic effects for the recipient bacteria that accept the transferred genes. Those receiving undesirable genetic traits are excluded generally from the bacterial population, and it becomes less likely that harmful genes are inherited by the next generation. However, mobile genetic elements (MGE) capable of self-transfer and self-replication often confer genetic traits that are advantageous to the recipient bacterial cells, and that can be propagated rapidly within the population [2]. HGT includes at least three types of mechanisms, including transformation, transduction, and conjugation [1]. In the event of transformation, recipient cells uptake foreign DNAs depending on their own genetic competence, and the foreign DNAs do not necessarily have a MGE function. In transduction, foreign DNAs are transferred proactively together by bacteriophage infection, but the infection itself is often harmful for the recipient cell [3]. Conjugation refers to the transfer of DNA to a recipient bacterium from a donor bacterium through a mating event. During conjugation, the DNA transfers through dedicated conjugation machinery, encoded by genes on the conjugative DNA element. Conjugative DNA elements may be either plasmids or conjugative transposons, which are now more commonly referred to as integrative and conjugative elements or ICEs. HGT is environmentally important, as it increases the diversity of bacterial communities; however, it also serves as a useful tool for engineering desired bacterial strains via recombinant DNA technology for research and industrial purposes.

The typical mechanism for conjugative plasmid transfer between two bacterial strains proceeds as follows [4–8]. First, in the donor, a specific enzyme, relaxase, cleaves a phosphodiester bond of the plasmid DNA to produce a nick at a specific site on its “origin of transfer,” or *oriT*. As a result, relaxase is covalently linked to the 5′-end of the single-stranded plasmid DNA at the nick, and a DNA–protein complex, called relaxosome, is formed. Next, another specific protein, the type IV coupling protein (T4CP), interacts with the relaxosome and targets it for transfer through a type IV secretion system from the donor to the recipient. When one unit of the plasmid DNA is transferred completely into the recipient cell, the single-stranded DNA is circularized and begins to replicate as maturation of the double-stranded circular plasmid DNA occurs in the transconjugant.

Generally speaking, plasmids are classified into two types: conjugative and non-conjugative ones. The former can transfer itself to the recipient using self-coding specific enzymes and a secretion system that is necessary for the transfer to occur; the latter cannot transfer itself because it lacks the required set of genes to do so. However, non-conjugative plasmids containing the *oriT* sequence, or mobilizable plasmids, can be mobilized by a co-resident conjugative plasmid within the same cell, in a phenomenon known as mobilization. Mobilizable plasmids are believed to need the *oriT* and its cognate *mob* gene, the gene encoding relaxase [9, 10], but recently it was discovered that relaxase is not necessary for plasmid mobilization if a plasmid carries a “mimic” of the *oriT* region found in conjugative plasmids [11].

pLS20 is a 65-kbp-conjugative plasmid isolated from a strain of *Bacillus subtilis* natto [12]. It can transfer itself between various *B. subtilis*-related Gram positive bacteria, including *Bacillus anthracis, Bacillus cereus*, *Bacillus licheniformis*, *Bacillus megaterium*, *Bacillus pumilus*, and *Bacillus thuringiensis* [13]. pLS20cat, a derivative of pLS20 carrying a chloramphenicol resistance gene [14–17], possesses the incredible quality of being able to very rapidly transfer itself between cells within 15 min by simply mixing the liquid cultures containing donor and recipient cells [18]. In addition, pLS20cat can function as a helper plasmid, with the ability to mobilize an independently replicating and co-resident mobilizable plasmid containing a short *oriT* sequence from pLS20cat (*oriT*_LS20_) that is unaccompanied by its cognate *mob* gene [19].

In this study, we inactivated the *oriT*_LS20_ region of pLS20cat to obtain pLS20catΔ*oriT*, rendering it completely immobile, but it was still able to facilitate mobilization of the plasmid containing the *oriT*_LS20_. Moreover, pLS20catΔ*oriT* was able to mobilize longer chromosomal DNA segments containing *oriT*_LS20_ independently of the natural competence of the recipient cell. The larger DNA mobilization was achieved after mixing the liquid cultures of the donor and recipient for only 15 min. This pLS20catΔ*oriT*-mediated chromosomal DNA mobilization will allow us to develop a novel genetic tool for rapid and repetitive accumulation of longer DNA segments into the recipient chromosome.

## Methods

### Bacterial strains and growth conditions

The bacterial strains and plasmids used in this study are listed in Table 1. Synthetic oligonucleotides used as PCR primers are shown in Table 2. Bacterial strains were grown on Lysogeny Broth (LB) medium (Difco) at 37 °C. When necessary, the medium was supplemented with antibiotics: 5 μg ml^−1^ chloramphenicol, 1 μg ml^−1^ erythromycin, 100 μg ml^−1^ spectinomycin, and 10 μg ml^−1^ kanamycin.

**Table 1:** Strains and plasmids used in this study

Strains and plasmids	Relevant genotype or description	Source or references
Strains		
*B. subtili*s		
PKS11	*trpC2* pLS20cat	[15]
GR138	*trpC2* pLS20cat pGR16B	[19]
TMO310	*trpC2 aprE*::(*spc lacI* P*spac*-*mazF*)	[20]
TMO311	*trpC2 aprE*::(*kan lacI* P*spac*-*mazF*)	[20]
YNB001	*trpC2 comK*::*spc*	This study
YNB022	*trpC2* pLS20cat (*kan lacI* P*spac*-*mazF)*	This study
YNB026	*trpC2* pLS20catΔ*oriT*	This study
YNB031	*trpC2* pLS20catΔ*oriT* pGR16B	This study
YNB060	*trpc2 aprE*::*kan yhfM*::(*oriT*_LS20_-F *erm*)	This study
YNB061	*trpc2 aprE*::*kan yhfM*::(*oriT*_LS20_-R *erm*)	This study
YNB069	*trpc2 aprE*::*kan yhfK*::(*oriT*_LS20_-F *erm*)	This study
YNB062	*trpc2 aprE*::*kan yhfC*::(*oriT*_LS20_-F *erm*)	This study
YNB097	*trpc2 aprE*::*kan yhcT*::(*oriT*_LS20_-F *erm*)	This study
YNB065	*trpc2 aprE*::*kan yhfM*::(*oriT*_LS20_-F *erm*) pLS20cat	This study
YNB066	*trpc2 aprE*::*kan yhfM*::(*oriT*_LS20_-R *erm*) pLS20cat	This study
YNB071	*trpc2 aprE*::*kan yhfK*::(*oriT*_LS20_-F *erm*) pLS20cat	This study
YNB067	*trpc2 aprE*::*kan yhfC*::(*oriT*_LS20_-F *erm*) pLS20cat	This study
YNB099	*trpc2 aprE*::*kan yhcT*::(*oriT*_LS20_-F *erm*) pLS20cat	This study
YNB091	*trpc2 aprE*::*kan yhfM*::(*oriT*_LS20_-F *erm*) pLS20catΔ*oriT*	This study
YNB095	*trpc2 aprE*::*kan yhfM*::(*oriT*_LS20_-R *erm*) pLS20catΔ*oriT*	This study
YNB092	*trpc2 aprE*::*kan yhfK*::(*oriT*_LS20_-F *erm*) pLS20catΔ*oriT*	This study
YNB094	*trpc2 aprE*::*kan yhfC*::(*oriT*_LS20_-F *erm*) pLS20catΔ*oriT*	This study
YNB100	*trpc2 aprE*::*kan yhcT*::(*oriT*_LS20_-F *erm*) pLS20catΔ*oriT*	This study
Plasmids		
pLS20cat	Conjugative plasmid pLS20 with a chloramphenicol resistance gene inserted in the unique *Sal*l site	[18]
pLS20catΔ*oriT*	pLS20cat without *oriT*_LS20_	This study
pGR16B	Mobilizable plasmid containing *oriT*_LS20_ and erythromycin resistance gene	[19]

**Table 2:** Oligonucleotides used in this study

Oligonucleotides	Sequences (5′→3′)
spc-F	GAGTCAGAAAACAGACGCATAAACGCTAACGGTCAGC
spc-R	CTAATACCGTTCCCCGAGAAGCTTCACTAAATTAAAGTAATAAAGC
comK-uF	AGAGCGTAAGAAACGCATC
comK-uR	TGCGTCTGTTTTCTGACTC
comK-dF	CTCGGGGAACGGTATTAG
comK-dR	CGAAGATCTGCCTACTGAAC
oriT-uF	TAAATAACATGACTGTGGAAATGAC
oriT-uR	GCTTGAGTCAATTCCGCTGTCGTTAGTCTTCGATGACGAGATTG
oriT-dF	CTGATTGGGTAGGATCCCCGAGAAAGAGCAATCTCGTCATCGAAGACTAAAAAAAGAAACACTTATTTGAACAGATC
oriT-dR	GCGTCTTCTTAAAACGCTG
mazF-F	CGACAGCGGAATTGACTCAAGC
mazF-R	CGGGGATCCTACCCAATCAG
oriT-F	AAAGAGCAATCTCGTCATCGAAGACTAAATTTC
oriT-R	TTGTTAACGCTCCTTTTCATCGATTTCTG
erm-F1	CAGAAATCGATGAAAAGGAGCGTTAACAAGAGTGTGTTGATAGTGCAGTATC
erm-F2	GAAATTTAGTCTTCGATGACGAGATTGCTCTTTGAGTGTGTTGATAGTGCAGTATC
erm-R	CTACATTCCCTTTAGTAACGTGTAAC
yhfM-uF	GATCGTGAAAGGCCCCAATGTG
yhfM-uR1	GAAATTTAGTCTTCGATGACGAGATTGCTCTTTGAAGCAAAGGATTGAAAATGAAAAAGCG
yhfM-uR2	CAGAAATCGATGAAAAGGAGCGTTAACAAGAAGCAAAGGATTGAAAATGAAAAAGCG
yhfM-dF	GTTACACGTTACTAAAGGGAATGTAGCACTATTTTTTTCATTTGCATCACTCCAAAC
yhfM-dR	ATCAGCGAAAGCACAAACACAAAACC
yhfK-uF	ATGATAAAATGACCACCGAAGAATTCCG
yhfK-uR1	GAAATTTAGTCTTCGATGACGAGATTGCTCTTTCACTTTCATGTGAATCCCTCCTGCC
yhfK-dF	GTTACACGTTACTAAAGGGAATGTAGGAAACTATGACAGTACTGACACTCAGGGC
yhfK-dR	GACGAGCTCAACCTTTGGCAGC
yhfC-uF	GCCAAATGGAGGCCGTATGTCAG
yhfC-uR1	GAAATTTAGTCTTCGATGACGAGATTGCTCTTTTGACCATTTTTCAGCCTCCTTTTTCTTTTTC
yhfC-dF	GTTACACGTTACTAAAGGGAATGTAGGATTGTAAAAGCAAAAAGGGTGTTTCAATAAAAGG
yhfC-dR	GGCTTGGGATCGATACAAGTTCTTTAATGAG
yhcT-uF	TTCGGGGACGAAAAATAGCACAGATC
yhcT-uR1	GAAATTTAGTCTTCGATGACGAGATTGCTCTTTCTGCTGATATGAAAAACCTTTGCCG
yhcT-dF	GTTACACGTTACTAAAGGGAATGTAGAGCCCTCTGCCTTTTTGGTTCATG
yhcT-dR	GCTTTGTTAGTCTTCTTTTGAAAGTCAGAAAAAGC

### Construction of the recipient strain

The *comK* gene of strain 168 was inactivated by replacement with a spectinomycin resistance gene, as follows. Two DNA fragments, each of which corresponded to upstream and downstream regions of *comK*, were amplified by PCR using 168 DNA as a template with primers comK-uF/comK-uR for the upstream fragment and comK-dF/comK-dR for the downstream fragment (Table 2). Another DNA fragment containing the spectinomycin resistance gene of strain TMO310 (Table 1) was amplified using primers spc-F/spc-R (Table 2). The three fragments were ligated together by recombinant PCR using primers comK-uF/comK-dR to sandwich the spectinomycin resistance gene between the upstream and downstream regions of *comK*. The recombinant PCR fragment was transformed into strain 168 conferring spectinomycin resistance and yielding the new strain, YNB001 (*comK*::*spc*), which was used as the recipient for the conjugative DNA transfer in this study.

### Construction of pLS20catΔ*oriT*

The *oriT*_LS20_ region of pLS20cat was inactivated by marker-free deletion, as previously described [20]. Two DNA fragments corresponding to the upstream (fragment 1) and downstream (fragment 2) regions of *oriT*_LS20_ were amplified by PCR using pLS20cat DNA as the template with primers oriT-uF/oriT-uR for the upstream region, and oriT-dF/oriT-dR for the downstream region (Table 2). Because the tail of fragment 1 and the head of fragment 2 were identical for 30 bp, those regions were responsible for the later deletion of the *oriT*_LS20_ region by intramolecular recombination. Another DNA fragment of the *mazF kan* cassette (fragment 3) was amplified from TMO311 DNA (Table 1) using primers mazF-F/mazF-R (Table 2). The three PCR fragments were designed to be connected in the order 1–3–2 by recombinant PCR using primers oriT-uF/oriT-dR. The recombinant PCR fragment was transformed then into strain PKS11 (Table 1) to confer kanamycin resistance, obtaining the new strain YNB022, in which pLS20cat was altered by integrating the PCR fragment through a double crossover event at the *oriT*_LS20_ region. YNB022 was grown overnight at 37 °C in LB liquid medium containing kanamycin. An aliquot of the culture was transferred into a fresh LB liquid medium containing 1 mM isopropyl-thiogalactopyranoside (IPTG) and the cells were allowed to grow for 2 h at 37 °C. Then, an aliquot of the culture was spread on an LB plate containing 1 mM IPTG and incubated at 37 °C overnight. In the presence of IPTG, *mazF* was expressed, producing a suicidal toxin so that only the cells that could pop-out the *mazF kan* cassette through intramolecular recombination could survive. Of those colonies appearing on the plate, kanamycin-sensitive colonies were sequenced to confirm the correct deletion of the *oriT*_LS20_ region. The resulting plasmid was designated as pLS20catΔ*oriT*.

### Construction of the donor strains

**Fig. 1:**
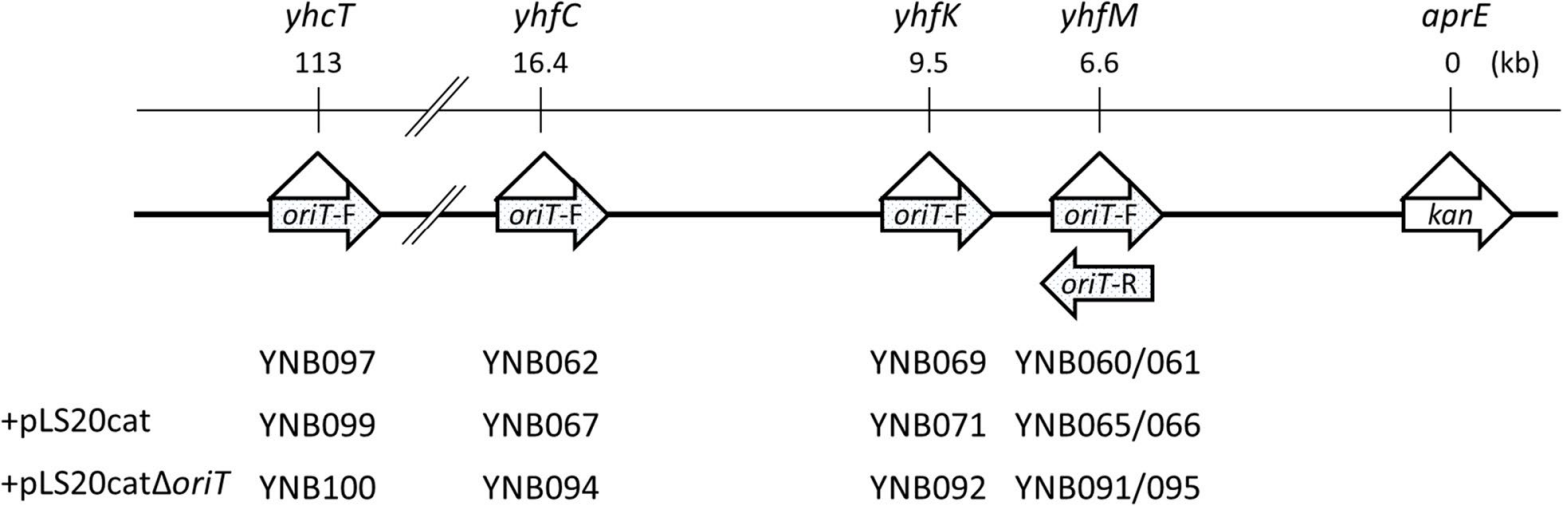
Schematic representation of the integration of the loci of *oriT*_LS20_ and the kanamycin resistance gene of the donor strains. The gene loci where *oriT*_LS20_ was integrated and the distances from the *aprE* loci where the kanamycin resistance gene (*kan*) was integrated are shown (top). The *oriT*_LS20_ regions in donor strains are shown with forward- (*oriT*-F) and reverse- (*oriT*-R) oriented arrowheads (middle). Names of strains are aligned underneath the corresponding integration loci of *oriT*_LS20_ (bottom)

The donor strain YNB060 was constructed as follows (Fig. 1). Two fragments corresponding to upstream (fragment 1) and downstream (fragment 4) regions of *yhfM* were amplified from 168 DNA using primers yhfM-uF/yhfM-uR1 (for upstream) and yhfM-dF/yhfM-dR (for downstream) (Table 2). Fragment 2 containing the *oriT*_LS20_ was amplified using pLS20cat as a template with primers oriT-F/oriT-R (Table 2). In addition, fragment 3 carrying the erythromycin resistance gene was amplified using plasmid pMutin2 as the template with primers erm-F1/erm-R (Table 2). Fragments 1–4 were ligated in the order 1–2–3–4 by recombinant PCR using primers yhfM-uF/yhfM-dR. Strain TMO311 (*aprE*::*kan*) was transformed with the recombinant PCR fragment to select colonies resistant both to erythromycin and kanamycin. The resulting strain was designated as YNB060 (Table 1), which had the erythromycin marker with *oriT*_LS20_ and kanamycin marker at both the *yhfM* and *aprE* loci, located 6.6 kb apart from each other on the same chromosome. In addition, the direction of replication of *oriT*_LS20_ was oriented toward the kanamycin marker located 6.6 kb downstream.

Another strain, YNB061, was constructed similarly as described above. Two fragments of *yhfM*, representing upstream and downstream regions, were amplified using primers yhfM-uF/yhfM-uR2 and yhfM-dF/yhfM-dR (Table 2), respectively. The *oriT*_LS20_ fragment (fragment 2) and the erythromycin resistance fragment (fragment 3) were amplified using primers oriT-F/oriT-R and erm-F2/erm-R (Table 2), respectively. The four fragments were ligated by recombinant PCR using primers yhfM-uF/yhfM-dR. Strain TMO311 was transformed with the recombinant PCR fragment selecting erythromycin-resistant colonies as YNB061 (Table 1). In contrast to YNB060, in YNB061 the direction of replication of *oriT*_LS20_ on the *yhfM* locus was oriented oppositely to the kanamycin marker on the *aprE* locus.

The other additional strains, YNB069, YNB062, and YNB097, were constructed similarly to those described above (Fig. 1). For YNB069, two fragments of upstream (fragment 1) and downstream (fragment 4) regions of *yhfK* were amplified from 168 DNA using primers yhfK-uF/yhfK-uR1 and yhfK-dF/yhfK-dR (Table 2), respectively. For YNB062, two fragments of upstream and downstream regions of *yhfC* were amplified using primers yhfC-uF/yhfC-uR1 and yhfC-dF/yhfC-dR (Table 2), respectively. For YNB097, two fragments of upstream and downstream regions of *yhcT* were amplified using primers yhcT-uF/yhcT-uR1 and yhcT-dF/yhcT-dR (Table 2), respectively. The *oriT*_LS20_ fragment (fragment 2) and the erythromycin resistance fragment (fragment 3) were the same as those used above for YNB060 construction. For each case, the respective four fragments were ligated by recombinant PCR using primers yhfK-uF/yhfK-dR for YNB069, primers yhfC-uF/yhfC-dR for YNB062, and primers yhcT-uF/yhcT-dR for YNB097 (Table 2). Each of the recombinant PCR fragments was used to transform TMO311 (*aprE*::*kan*) to select colonies resistant to both erythromycin and kanamycin. The resulting strains were designated as YNB069, YNB062, and YNB097 (Table 1), which all had the erythromycin marker with *oriT*_LS20_ at the *yhfK*, *yhfC*, and *yhcT* loci, and the kanamycin marker at the *aprE* locus set apart from each other by 9.5, 16.4, and 113 kb within the chromosome, respectively. In addition, in all these strains, the direction of replication of *oriT*_LS20_ was forward-oriented to the kanamycin marker.

### Conjugative DNA mobilization

Conjugative DNA mobilization was performed in the liquid medium, as previously described [18]. Donor and recipient strains were cultured independently overnight in 5 ml of LB liquid medium containing the appropriate antibiotics at 37 °C with shaking at 180 rpm. Each of the cultures was diluted to an optical density for the cell of 0.05 at 600 nm (OD_600_) in 5 ml of fresh LB medium without antibiotics and incubated at 37 °C with shaking at 180 rpm. When OD_600_ reached 0.5–0.7, 500 μl of the donor and recipient cultures were mixed in a 1.5 ml microtube to stand at 37 °C for 15 min. The mixture was serially diluted and spread on LB plates containing various combinations of antibiotics to grow colonies overnight. On their respective plates, colonies were counted as colony forming units (CFU) of transformed recipients produced by conjugative transfer and mobilization (transconjugants) to calculate mobilization efficiencies [CFU of transconjugants/CFU of total recipients × 10^6^ (ppm)].

## Results

### pLS20catΔ*oriT* cannot transfer itself but can help to mobilize a co-resident plasmid carrying *oriT*_LS20_

**Fig. 2:**
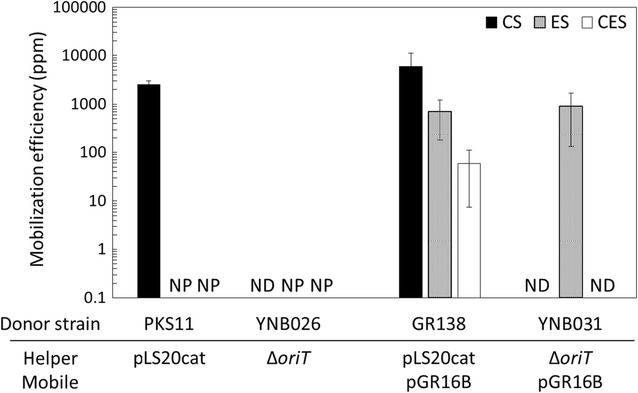
Mobilization efficiencies of the mobilizable plasmid, pGR16B, and the helper plasmids, pLS20cat and pLS20catΔ*oriT*. Liquid cultures of the recipient strain YNB001 (*comK*::*spc*) and one of the donor strains: PKS11 (168 with pLS20cat), YNB026 (168 with pLS20catΔ*oriT*), GR138 (168 with pLS20cat and pGR16B), and YNB031 (168 with pLS20catΔ*oriT* and pGR16B), were mixed for conjugative transfer and spread on LB plates containing both chloramphenicol and spectinomycin (CS), both erythromycin and spectinomycin (ES), chloramphenicol, erythromycin, and spectinomycin (CES), and spectinomycin alone. Colonies were counted as CFUs to calculate mobilization efficiencies [CFU of transconjugants (colonies on the CS, ES, and CES plates)/CFU of total recipients (colonies on the spectinomycin plate) × 10^6^ (ppm)]. Values are means with standard deviations from three independent experiments. *ND* not detected (< 0.01 ppm). *NP* not performed

As previously described [14–16], pLS20cat has the complete set of genes required for its own conjugative transfer. In fact, pLS20cat transfers itself from the donor PKS11 (Table 1, 168 with pLS20cat) or GR138 (strain 168 with pLS20cat and pGR16B) to the recipient YNB001 (*comK*::*spc*) within only 15 min after mixing the two parental liquid cultures, resulting in a large number (more than 2500 ppm) of recipient cells with acquired chloramphenicol resistance appearing as transconjugants (Fig. 2). It is also known that pLS20cat is capable of mobilizing a co-resident plasmid, pGR16B, carrying *oriT*_LS20_ and erythromycin resistance gene [19], as we observed the donor, GR138, confer erythromycin resistance on nearly 1000 ppm of recipient cells (Fig. 2). These results imply that the helper pLS20cat could be nearly twice more efficient at transforming recipient cells than the mobilizable plasmid pGR16B. In addition, about 100 ppm of the recipients obtained resistance to both erythromycin and chloramphenicol (Fig. 2), suggesting that about 10% of the transconjugants that accepted pGR16B also may have acquired pLS20cat.

Since the bacterial cells carrying pLS20cat do not accept the pLS20cat-mediated genetic transfer [14–16], the transconjugants that have accepted pLS20cat could not be transformed again using the same conjugative transfer system. On the other hand, the cells carrying pLS20cat could transfer not only pLS20cat itself but also mobilize co-resident pGR16B to other strains further. If these transconjugants were released into the environment, the antibiotic resistance genes would be spread to other bacterial cells, causing the undesirable emergence of new antibiotic-resistant bacteria [21, 22]. To avoid the self-transfer of pLS20cat, we aimed at knocking-out *oriT*_LS20_ in pLS20cat to construct pLS20catΔ*oriT*. As expected, the donor YNB026 did not transfer pLS20catΔ*oriT* at all, whereas YNB031 mobilized the co-resident pGR16B to confer erythromycin resistance on the recipients (Fig. 2). Furthermore, the mobilization efficiency of pGR16B was nearly the same whether pLS20cat or pLS20catΔ*oriT* served as the helper plasmid. These results indicate that the pLS20cat-dependent mobilization of pGR16B did not require self-mobility of the helper plasmid, pLS20cat. In addition, knocking-out *oriT*_LS20_ in pLS20cat did not affect the mobilization efficiency of the co-resident, pGR16B.

### pLS20catΔ*oriT* can mobilize chromosomal DNA containing *oriT*_LS20_

As shown above and in previous studies [19], pLS20cat can efficiently mobilize the co-resident mobilizable plasmid with *oriT*_LS20_ but without its cognate *mob* gene. Recently, conjugative transfer was shown to mobilize a large DNA fragment, representing the entire chromosome of *Mycoplasma* [23]. Thus, we conceived the idea that pLS20cat may be able to mobilize chromosomal DNA, depending on the status of the *oriT*_LS20_ region.

In the donor chromosome, *oriT*_LS20_ was introduced at the *yhfM* locus, 6.6 kb upstream of the kanamycin resistance gene at the *aprE* locus; in strains YNB060 and YNB061 (Table 1), the direction of replication of *oriT*_LS20_ was forward- and reverse-oriented to the kanamycin resistance gene, respectively. pLS20cat or pLS20catΔ*oriT* was introduced into the donor as the helper plasmid, yielding these new strains: (1) YNB065 (YNB060 with pLS20cat), (2) YNB066 (YNB061 with pLS20cat), (3) YNB091 (YNB060 with pLS20catΔ*oriT*), and (4) YNB095 (YNB061 with pLS20catΔ*oriT*). On the other hand, in the recipient strain YNB001, *comK* encoding the key transcription factor for natural competence was inactivated so that the strain completely lost its natural competence (data not shown).

**Fig. 3:**
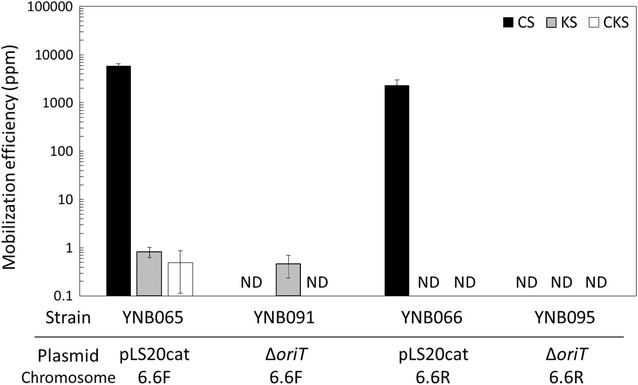
Mobilization efficiencies of the kanamycin resistance gene at the *aprE* locus and the helper plasmids, pLS20cat and pLS20catΔ*oriT*. YNB060 and YNB061 have *oriT*_LS20_ forward- and reverse-oriented to the kanamycin resistance gene (6.6F and R, respectively). Liquid cultures of the recipient strain YNB001 (*comK*::*spc*) and one of the donor strains: YNB065 (YNB060 with pLS20cat), YNB091 (YNB060 with pLS20catΔ*oriT*), YNB066 (YNB061 with pLS20cat), YNB095 (YNB061 with pLS20catΔ*oriT*), were mixed for conjugative transfer and spread on LB plates containing both chloramphenicol and spectinomycin (CS), both kanamycin and spectinomycin (KS), chloramphenicol, kanamycin, and spectinomycin (CKS), and spectinomycin alone. Colonies were counted as CFUs to calculate mobilization efficiencies [CFU of transconjugants (colonies on the CS, KS, and CKS plates)/CFU of total recipients (colonies on the spectinomycin plate) × 10^6^ (ppm)]. Values are means with standard deviations from three independent experiments. *ND* not detected (< 0.01 ppm)

Strains YNB065 and YNB066, both carrying pLS20cat, conferred chloramphenicol resistance on more than 2300 ppm of the recipients (Fig. 3), but YNB065 was able to confer kanamycin resistance on only 1 ppm of the recipient cells; YNB066 did not confer kanamycin resistance at all (Fig. 3). These results indicate that pLS20cat could transfer the kanamycin resistance gene located 6.6 kb downstream of *oriT*_LS20_, if the direction of *oriT*_LS20_ replication was forward-oriented to the kanamycin resistance gene. In addition, since the recipients had no natural competence, the acquisition of kanamycin resistance depended solely on the conjugative transfer. On the other hand, YNB065 conferred not only kanamycin resistance but also chloramphenicol resistance, on nearly 1 ppm of the recipients (Fig. 3). These results suggest that a large majority of the kanamycin-resistant recipients that accepted the chromosomal DNA could additionally have acquired the helper plasmid, pLS20cat.

When pLS20catΔ*oriT* was introduced as the helper plasmid into YNB060 (YNB091) and YNB061 (YNB095), it appeared that no recipient acquired chloramphenicol resistance (Fig. 3), confirming the loss of self-mobility in pLS20catΔ*oriT*. On the other hand, and more importantly, when YNB091 was used as the donor, pLS20catΔ*oriT* transferred kanamycin resistance to recipients with a similar efficiency and *oriT*_LS20_-orientation dependency to pLS20cat (Fig. 3). These results clearly indicate that pLS20catΔ*oriT* can exert its helper activity as efficiently as the original helper plasmid, pLS20cat, not only in the case of mobilizing pGR16B but also when mobilizing the chromosomal DNA. YNB095 did not confer kanamycin resistance on the recipients, confirming that the DNA mobilization depended on the forward-oriented *oriT*_LS20_.

**Fig. 4:**
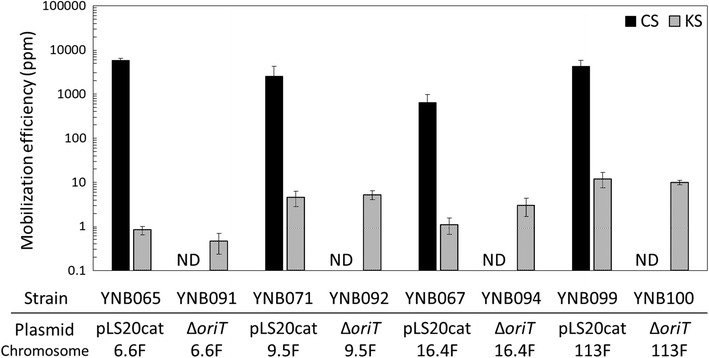
Mobilization efficiencies of the kanamycin resistance gene at the *aprE* locus and the helper plasmids, pLS20cat and pLS20catΔ*oriT*. Liquid cultures of the recipient strain YNB001 (*comK*::*spc*) and one of the donor strains: YNB065 (YNB060 with pLS20cat), YNB091 (YNB060 with pLS20catΔ*oriT*), YNB071 (YNB069 with pLS20cat), YNB092 (YNB069 with pLS20catΔ*oriT*), YNB067 (YNB062 with pLS20cat), YNB094 (YNB062 with pLS20catΔ*oriT*), YNB099 (YNB097 with pLS20cat), and YNB100 (YNB097 with pLS20catΔ*oriT*), were mixed for conjugative transfer of long DNA segments (6.6F, 9.5F, 16.4F, and 113F for 6.6, 9.5, 16.4, and 113 kb between *oriT*_LS20_ and the kanamycin marker, respectively), and spread on LB plates containing both chloramphenicol and spectinomycin (CS), both kanamycin and spectinomycin (KS), and spectinomycin alone. Colonies were counted as CFUs to calculate mobilization efficiencies [CFU of transconjugants (colonies on the CS and KS plates)/CFU of total recipients (colonies on the spectinomycin plate) × 10^6^ (ppm)]. Values are means with standard deviations from three independent experiments. *ND* not detected (< 0.01 ppm)

The distance between *oriT*_LS20_ and the kanamycin marker was extended by 9.5 kb, 16.4 kb, and 113 kb in a stepwise fashion in strains YNB069, YNB062, and YNB097, respectively (Fig. 1). In all of these strains, the direction of replication of *oriT*_LS20_ was forward-oriented to the kanamycin resistance gene. A helper plasmid, either pLS20cat or pLS20catΔ*oriT*, was introduced into each donor to create new donor strains: YNB071 (YNB069 with pLS20cat), YNB067 (YNB062 with pLS20cat), YNB099 (YNB097 with pLS20cat), YNB092 (YNB069 with pLS20catΔ*oriT*), YNB094 (YNB062 with pLS20catΔ*oriT*), and YNB100 (YNB097 with pLS20catΔ*oriT*). All donors with pLS20cat conferred chloramphenicol resistance on more than 600 ppm of recipient cells, whereas the other donors, with pLS20catΔ*oriT*, did not confer chloramphenicol resistance at all (Fig. 4). However, and more importantly, all of the strains with pLS20catΔ*oriT* were able to confer kanamycin resistance on 0.5–10.0 ppm of the recipient cells. Efficiencies were nearly equivalent to those achieved with YNB091 as the donor (Fig. 4), indicating that the length of mobilized DNA could be extended at least to 113 kb. In addition, pLS20catΔ*oriT* exhibited similar efficiencies to pLS20cat when mobilizing longer segments of chromosomal DNA (Fig. 4). These results also indicate that self-mobility of pLS20cat was not necessary for its helper function for mobilizing longer segments of chromosomal DNA.

## Discussion

Strains of *B. subtilis* 168 derivatives have an advantage over other bacteria because their natural competence and high recombination efficiency allow for plasticity of their genome. A number of artificial introductions and compilations of various sizes and kinds of DNA segments have been performed successfully into the *B. subtilis* genome [24]. Therefore, *B. subtilis* has been regarded, generally, as one of the most promising platforms for designing, assembling, and modifying synthetic DNA, or even on larger scales with an entire synthetic genome. Accordingly, there is increasing demand of novel genetic tools for mobilizing longer DNA segments, which will push forward the research and development in synthetic genome approaches [25].

Here we demonstrated that pLS20cat conjugative transfer was capable of mobilization of not only a mobilizable plasmid carrying *oriT*_LS20_ but also chromosomal DNA. In this study, however, both the donors and recipients were derived from the same parental strain, *B. subtilis* 168, and recombination events between the chromosome and the mobilized DNA could occur at any homologous locations; therefore, we are not able to state that the entire length of mobilized DNA accurately replaced the respective part of the chromosome. Nevertheless, we can assume, at least, that the mobilization of chromosomal DNA initiated at the integration point of *oriT*_LS20_ and continued until the kanamycin marker because mobilization was seen only when the direction of replication of *oriT*_LS20_ was forward-oriented to the kanamycin resistance gene (Fig. 3). Furthermore, the present results indicate that DNA segments up to 113 kb could be mobilized (Fig. 4), which may be one of the longest segments of DNA mobilized artificially from one cell to another within such a short period of only 15 min. For further applications, it would be worthwhile to test various conditions in order to extend the length of mobilizable DNA.

As mentioned above, the mobilization of chromosomal DNA depended upon the forward-orientation of the *oriT*_LS20_ replication region, implying that the replication origin may function unidirectionally; however, a previous study suggested the possibility that *oriT*_LS20_ may be able to replicate bidirectionally [19]. If this is true, it is likely that *oriT*_LS20_ replication in the reverse direction would be too weak to enable the mobilization of longer DNA segments. On the other hand, the reverse-oriented *oriT*_LS20_ could lead the counterclockwise replication of the other side of chromosome, which requires synthesis of much longer DNA to encounter the kanamycin marker. As we failed to detect any kanamycin-resistant transformant using the reverse-oriented *oriT*_LS20_, there could be a certain limit in the length of mobilizable DNA, which is yet to be defined.

We inactivated the *oriT*_LS20_ of pLS20cat to make pLS20catΔ*oriT*, which never transferred itself between cells but was able to mobilize longer segments of chromosomal DNA with nearly the same efficiency as the self-transfer of pLS20cat. To our knowledge, this is the first demonstration that the *oriT* function of pLS20cat is not required for performing its helper function in mobilizing DNA fragments containing *oriT*_LS20_. The DNA mobilization by the pLS20cat conjugative system only occurs to a recipient not harboring pLS20cat [14–16]. As described above, when donors with pLS20cat were used, more than 2000 ppm of the recipient cells became chloramphenicol resistant by accepting pLS20cat. On the other hand, the transfer of kanamycin resistance was seen for only 1–10 ppm of recipients. These results imply that nearly all the recipients that acquired the chromosomal DNA also could have accepted pLS20cat. Therefore, recipients that previously acquired chromosomal DNA with the help of pLS20cat could no longer accept a conjugative DNA mobilization based on the pLS20 system. On the other hand, recipients that acquired chromosomal DNA with the help of pLS20catΔ*oriT* did not have pLS20catΔ*oriT* and could accept new rounds of DNA mobilization. This acquired knowledge will be beneficial for developing a novel genetic tool for repetitive accumulation of longer DNA segments into the recipient chromosome. Furthermore, the pLS20catΔ*oriT* also would be useful for transforming other *B. subtilis*-related Gram positive bacteria, including *B. anthracis, B. cereus*, *B. licheniformis*, *B. megaterium*, *B. pumilus*, and *B. thuringiensis* [13].

## Conclusions

In this study, the *oriT*_LS20_ region of pLS20cat was eliminated to obtain pLS20catΔ*oriT*, which resulted in completely eliminating the plasmid’s own mobility, while maintaining an ability to efficiently mediate the conjugative mobilization of a neighboring mobilizable plasmid. Moreover, pLS20catΔ*oriT* was able to mobilize longer DNA segments, up to 113 kb of chromosomal DNA, containing the *oriT*_LS20_ region after mixing the liquid cultures of the donor and recipient for only 15 min. Understanding this chromosomal DNA mobilization by pLS20catΔ*oriT* will allow us to develop a novel genetic tool for the rapid, easy, and repetitive accumulation of longer DNA segments into a recipient chromosome.

## Abbreviations

CFU: colony forming units
HGT: horizontal gene transfer
IPTG: isopropyl-thiogalactopyranoside
LB: Lysogeny Broth
MGE: mobile genetic elements
T4CP: type IV coupling protein
PCR: polymerase chain reaction

## References

[1] Frost LS, Leplae R, Summers AO, Toussaint A (2005). Mobile genetic elements: the agents of open source evolution. Nat Rev Microbiol.

[2] Thomas CM, Nielsen KM (2005). Mechanisms of, and barriers to, horizontal gene transfer between bacteria. Nat Rev Microbiol.

[3] Davison J (1999). Genetic exchange between bacteria in the environment. Plasmid.

[4] Abajy MY, Kopeć J, Schiwon K, Burzynski M, Döring M, Bohn C (2007). A type IV-secretion-like system is required for conjugative DNA transport of broad-host-range plasmid pIP501 in Gram-positive bacteria. J Bacteriol.

[5] Alvarez-Martinez CE, Christie PJ (2009). Biological diversity of prokaryotic type IV secretion systems. Microbiol Mol Biol Rev.

[6] Grohmann E, Muth G, Espinosa M (2003). Conjugative plasmid transfer in Gram-positive bacteria. Microbiol Mol Biol Rev.

[7] Wallden K, Rivera-Calzada A, Waksman G (2010). Type IV secretion systems: versatility and diversity in function. Cell Microbiol.

[8] Zechner EL, Lang S, Schildbach JF (2012). Assembly and mechanisms of bacterial type IV secretion machines. Philos Trans R Soc B.

[9] Francia MV, Varsaki A, Garcillán-Barcia MP, Latorre A, Drainas C, De La Cruz F (2004). A classification scheme for mobilization regions of bacterial plasmids. FEMS Microbiol Rev.

[10] Smillie C, Garcillan-Barcia MP, Francia MV, Rocha EPC, de la Cruz F (2010). Mobility of plasmids. Microbiol Mol Biol Rev.

[11] Ramsay JP, Firth N (2017). Diverse mobilization strategies facilitate transfer of non-conjugative mobile genetic elements. Curr Opin Microbiol.

[12] Tanaka T, Kuroda M, Sakaguchi K (1977). Isolation and characterization of four plasmids from *Bacillus subtilis*. J Bacteriol.

[13] Koehler TM, Thorne CB (1987). *Bacillus subtilis* (*natto*) plasmid pLS20 mediates interspecies plasmid transfer. J Bacteriol.

[14] Meijer WJJ, Boer AJ, Tongeren SV, Venema G, Bron S (1995). Characterization of the replication region of the *Bacillus subtilis* plasmid pLS20: a novel type of replicon. Nucleic Acids Res.

[15] Singh PK, Ramachandran G, Durán-Alcalde L, Alonso C, Wu LJ, Meijer WJJ (2012). Inhibition of *Bacillus subtilis* natural competence by a native, conjugative plasmid-encoded *comK* repressor protein. Environ Microbiol.

[16] Singh PK, Ramachandran G, Ramos-Ruiz R, Peiro-Pastor R, Abia D, Wu LJ (2013). Mobility of the native *Bacillus subtilis* conjugative plasmid pLS20 is regulated by intercellular signaling. PLoS Genet.

[17] Ramachandran G, Singh PK, Luque-Ortega JR, Yuste L, Alfonso C, Rojo F, Wu LJ, Meijer WJJ (2014). A complex genetic switch involving overlapping divergent promoters and DNA looping regulates expression of conjugation genes of a Gram-positive plasmid. PLoS Genet.

[18] Itaya M, Sakaya N, Matsunaga S, Fujita K, Kaneko S (2006). Conjugational transfer kinetics of pLS20 between *Bacillus subtilis* in liquid medium. Biosci Biotechnol Biochem.

[19] Ramachandran G, Miguel-Arribas A, Abia D, Singh PK, Crespo I, Gago-Córdoba C (2017). Discovery of a new family of relaxases in Firmicutes bacteria. PLoS Genet.

[20] Morimoto T, Ara K, Ozaki K, Ogasawara N (2009). A new simple method to introduce marker-free deletions in the *Bacillus subtilis* genome. Genes Genet Syst..

[21] Davies J, Davies D (2010). Origins and evolution of antibiotic resistance. Microbiol Mol Biol Rev.

[22] Berglund B (2015). Environmental dissemination of antibiotic resistance genes and correlation to anthropogenic contamination with antibiotics. Infect Ecol Epidemiol..

[23] Dordet-Frisoni E, Sagné E, Baranowski E, Breton M, Nouvel LX, Blanchard A (2014). Chromosomal transfers in mycoplasmas: when minimal genomes go mobile. MBio..

[24] Itaya M, Fujita K, Kuroki A, Tsuge K (2008). Bottom-up genome assembly using the *Bacillus subtilis* genome vector. Nat Methods.

[25] Richardson SM, Mitchell LA, Stracquadanio G, Yang K, Dymond JS, DiCarlo JE (2017). Design of a synthetic yeast genome. Science.

